# Negative effects on medical students’ scores for clinical performance during the COVID-19 pandemic in Taiwan: a comparative study

**DOI:** 10.3352/jeehp.2023.20.37

**Published:** 2023-12-26

**Authors:** Eunice Jia-Shiow Yuan, Shiau-Shian Huang, Chia-An Hsu, Jiing-Feng Lirng, Tzu-Hao Li, Chia-Chang Huang, Ying-Ying Yang, Chung-Pin Li, Chen-Huan Chen

**Affiliations:** 1 Department of Medical Education, Taipei Veterans General Hospital, Taipei, Taiwan; 2 Department of Family Medicine, Taipei Veterans General Hospital, Taipei, Taiwan; 3 School of Medicine, College of Medicine, National Yang Ming Chiao Tung University, Taipei, Taiwan; 4 School of Public Health, National Defense Medical Center, Taipei, Taiwan; 5 Department of Ophthalmology, Taipei Veterans General Hospital, Taipei, Taiwan; 6 Department of Radiology, Taipei Veterans General Hospital, Taipei, Taiwan; 7 Division of Allergy, Immunology, and Rheumatology, Department of Internal Medicine, Shin Kong Wu Ho-Su Memorial Hospital, Taipei, Taiwan; 8 Division of Clinical Skills Training, Department of Medical Education, Taipei Veterans General Hospital, Taipei, Taiwan; 9 Clinical Innovation Center, Department of Medical Education, Taipei Veterans General Hospital, Taipei, Taiwan; 10 Division of Gastroenterology and Hepatology, Department of Medicine, Taipei Veterans General Hospital, Taipei, Taiwan; 11 Therapeutic and Research Center of Pancreatic Cancer, Taipei Veterans General Hospital, Taipei, Taiwan; Hallym University, Korea

**Keywords:** COVID-19, Curriculum, Medical education, Medical student, Taiwan

## Abstract

**Purpose:**

Coronavirus disease 2019 (COVID-19) has heavily impacted medical clinical education in Taiwan. Medical curricula have been altered to minimize exposure and limit transmission. This study investigated the effect of COVID-19 on Taiwanese medical students’ clinical performance using online standardized evaluation systems and explored the factors influencing medical education during the pandemic.

**Methods:**

Medical students were scored from 0 to 100 based on their clinical performance from 1/1/2018 to 6/31/2021. The students were placed into pre-COVID-19 (before 2/1/2020) and midst-COVID-19 (on and after 2/1/2020) groups. Each group was further categorized into COVID-19-affected specialties (pulmonary, infectious, and emergency medicine) and other specialties. Generalized estimating equations (GEEs) were used to compare and examine the effects of relevant variables on student performance.

**Results:**

In total, 16,944 clinical scores were obtained for COVID-19-affected specialties and other specialties. For the COVID-19-affected specialties, the midst-COVID-19 score (88.51–3.52) was significantly lower than the pre-COVID-19 score (90.14–3.55) (P<0.0001). For the other specialties, the midst-COVID-19 score (88.32–3.68) was also significantly lower than the pre-COVID-19 score (90.06–3.58) (P<0.0001). There were 1,322 students (837 males and 485 females). Male students had significantly lower scores than female students (89.33–3.68 vs. 89.99–3.66, P=0.0017). GEE analysis revealed that the COVID-19 pandemic (unstandardized beta coefficient=-1.99, standard error [SE]=0.13, P<0.0001), COVID-19-affected specialties (B=0.26, SE=0.11, P=0.0184), female students (B=1.10, SE=0.20, P<0.0001), and female attending physicians (B=-0.19, SE=0.08, P=0.0145) were independently associated with students’ scores.

**Conclusion:**

COVID-19 negatively impacted medical students' clinical performance, regardless of their specialty. Female students outperformed male students, irrespective of the pandemic.

## Graphical abstract

[Fig f5-jeehp-20-37]

## Introduction

### Background/rationale

Coronavirus disease 2019 (COVID-19) has spread dramatically worldwide, with more than 700 million confirmed cases and over 6 million deaths as of June 2023, according to the World Health Organization COVID-19 Dashboard [1]. Beginning in February 2020, the COVID-19 pandemic has led to training hospitals offering fewer or suspended opportunities for hands-on clinical participation by medical students to ensure their safety in the United States [2]. Medical education in Taiwan, accordingly, has become vastly different from what was traditionally offered prior to the pandemic, creating unfamiliarity and challenges as well as leading to a “new normal.”

Generally, the assessment tools implemented in Taiwan follow the 6 domains of competencies as established by the Accreditation Council for Graduate Medical Education model: (1) medical knowledge, (2) patient care, (3) professionalism, (4) communication, (5) interpersonal skills, and (6) practice-based learning and improvement [3]. While the pandemic has led to the segregation of medical students from regular hospital training owing to transmission concerns, the assessment of medical students has persisted to assist students in identifying and responding to their individual learning needs. Notably, technology has been adopted to ensure the continuation of medical education for curriculum delivery, case presentation, inpatient care, and outpatient consultation during the pandemic worldwide [2,4]. Most in-person training has transitioned to virtual learning to reduce the number of people physically getting together in one place. This has provided learning opportunities regardless of the whereabouts and safety of the medical students.

Major infectious disease epidemics have been shown to increase public fear, with decreased utilization of ambulatory services, inpatient care, and emergency care in Taiwan [5]. The diminishing patient population may also significantly hinder medical students’ learning experiences in most specialties. Despite fewer patients in most departments, certain specialties, such as emergency, pulmonary, and infectious Medicine, are heavily burdened by more critically ill patients. This dire situation has created another set of challenges for trainers and trainees. Many studies have highlighted the differences between male and female students in coping with stressful situations in the United States [6]. Based on previous articles, we argue that while COVID-19 poses a significant physical and mental health burden for health professionals and medical students, specialty and gender are 2 factors that appear to play a significant role in accommodating learning capabilities.

### Objectives

In Taiwan, COVID-19 has undeniably limited traditional educational methods given the newly established governmental regulations on social distancing. Students are likely to be affected by risk mitigation in addition to workforce and resource utilization. The pandemic has disrupted the traditional education routines of future medical professionals. This study aimed to evaluate the impact of COVID-19 on the clinical performance of Taiwanese medical students by comparing students’ scores before and during the COVID-19 pandemic using standardized assessment tools and to explore the factors associated with the efficacy of medical education within the scoring systems.

## Methods

### Ethics statement

This study was approved by the Institutional Review Board of Taipei Veterans General Hospital (2022-01-021CC) and the requirement for informed consent was waived.

### Study design

This is a 2-group comparative study examining students’ scores before and during the COVID-19 pandemic according to the Strengthening the Reporting of Observational Studies in Epidemiology statement available from: https://www.strobe-statement.org.

### Clinical training background

**Fig. 1.**
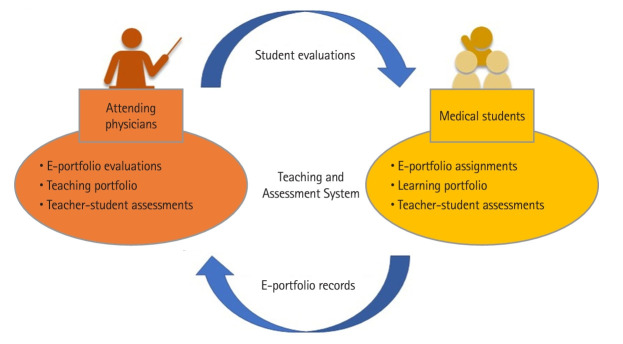
E-portfolio. The students receive both in-hospital rotations and in-class lectures during the clinical years. Taipei Veterans General Hospital clerkship mandates that students rotate through various surgical specialties, internal medicine specialties, diagnostic medicine, psychiatry, and public health. The attending physicians assessed the students’ monthly clinical performances using an online student passport, the electronic portfolio.

The medical education program in Taiwan is a 6-year curriculum, in which the last 2 years are clinical years. Students receive both in-hospital rotations and lectures during their clinical experience. The Taipei Veterans General Hospital clerkship mandates that students rotate through various surgical, internal medicine, diagnostic medicine, psychiatry, and public health specialties. Attending physicians assess students’ monthly clinical performance using an online student passport, the electronic portfolio (e-portfolio) as shown in Fig. 1. Before COVID-19, medical students were divided into teams with one or 2 attending physicians as leaders, and they learned to work as front-line physicians for patient care, and assist attending physicians and resident physicians during their clinical practice. The Taipei Veterans General Hospital medical student education system was affected during COVID-19, and compromises were made, including an alternative for the delivery of medical education and testing, the redeployment of personnel, and decreased routine patient cases.

### Setting

The study used a before-after (COVID-19) comparative study design that collected all medical students’ clinical scores from Taipei Veterans General Hospital student passports from 1/1/2018 to 6/31/2021. Scores before 2/1/2020 were placed in the pre-COVID-19 group, and scores from and after 2/1/2020 were placed in the midst-COVID-19 group. Each group was further categorized into COVID-19-affected specialties (pulmonary, infectious, and emergency medicine) and other specialties.

### Participants

Taipei Veterans General Hospital medical students’ e-portfolio scores provided by attending physicians were collected and divided by gender. The scores were further divided into COVID-19-affected specialties (emergency, pulmonary, and infectious medicine) and other specialties. All 830 students received a clinical performance score from 0 to 100 at the end of each rotation in internal medicine, surgical medicine, and other specialties.

### Variables

The scores of different specialties and pre- and midst-COVID-19 differences were used to assess the performance of the examinees in this study. The maximum score for each specialty was 100. Differences were compared between COVID-19-affected specialties and the other specialties, between pre- and midst-COVID-19 periods, according to students’ age, and according to the gender of students and attending physicians.

### Data sources/measurement

The examiners scored the students’ performance using a computer program and the results were automatically processed. All variables were recorded in an Excel spreadsheet (Microsoft Corp.).

### Bias

No selection bias was found in the study design.

### Study size

Because all medical students were included, prior sample size estimation was not performed. Post-hoc power analysis for the score differences between pre-COVID-19 group and the midst-COVID-19 group showed a power (1-β error probability) of more than 0.9999 in both COVID-19-affected specialties and other specialties. Post hoc power analysis was done with an α of 0.05 and a 2-tailed test using G*Power ver. 3.1.9.4 (Heinrich-Heine-Universität Düsseldorf).

### Statistical methods

This study analyzed the differences in performance among participants educated in different specialties before and during the COVID-19 pandemic. The chi-square test and 2-sample t-test were used to analyze demographic and score differences, respectively. Cohen’s d was utilized to report the effect size of differences. Generalized estimating equations (GEEs) were used to evaluate the impact of student’s age, student’s gender, attending physician’s gender, specialty attended, and the COVID-19 pandemic on students’ scores. Statistical analyses were performed using SAS ver. 9.3 (SAS Institute Inc.) and G*Power. Statistical significance was set at a P-value <0.05.

## Results

### Participants

Table 1 shows the baseline characteristics of the participants. This study included 1,322 students and 918 attending physicians. In total, 16, 944 clinical scores of these students were collected from the online passport system. Clinical scores were divided into 2 subgroups: pre-COVID-19 (n=1,434) and midst-COVID-19 (n=598). The other specialties group was divided into 10,494 scores in the pre-COVID-19 subgroup and 4,418 in the midst-COVID-19 subgroup.

### Main results

**Fig. 2.**
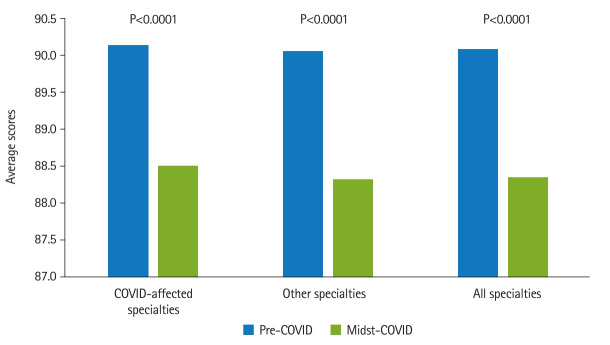
Pre-COVID-19 and midst-COVID-19 score comparison. Within the COVID-19-affected specialties group, the pre-COVID-19 group scored 90.14–3.55, and the midst-COVID-19 group scored 88.51–3.52 (Cohen’s d=0.33, P<0.0001). Within the other specialties group, the pre-COVID-19 group scored 90.06–3.58, and the midst-COVID-19 group scored an average of 88.32–3.68 (Cohen’s d=0.34, P<0.0001). COVID-19, coronavirus disease 2019.

**Fig. 3.**
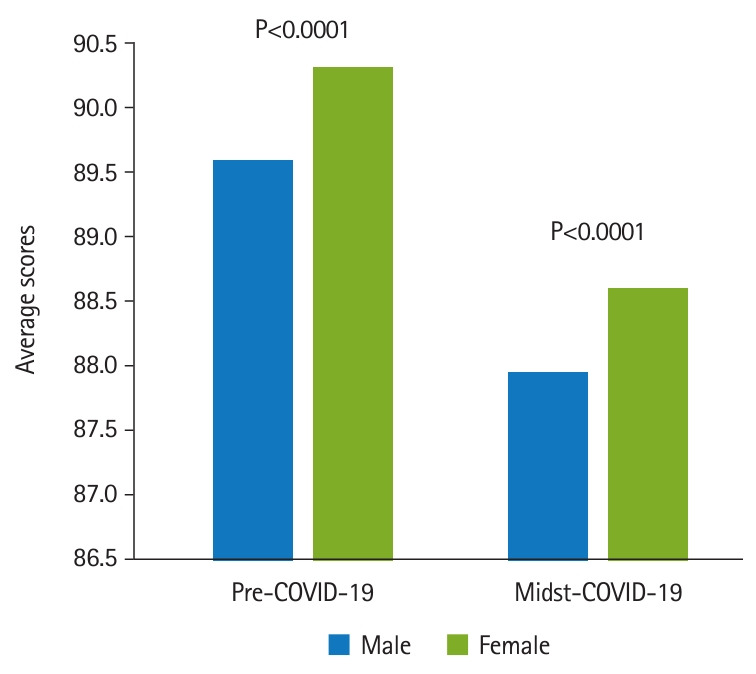
Male attending physician grading: male students versus female students. Pre-COVID-19 male students received an average of 89.61–3.65, and the female students received an average of 90.32–3.94 (Cohen’s d=0.13, P<0.0001). The midst-COVID-19 male students received an average of 87.9–3.69 and the female students received an average of 88.61–3.79 (Cohen’s d=0.13, P<0.0001). COVID-19, coronavirus disease 2019.

**Fig. 4.**
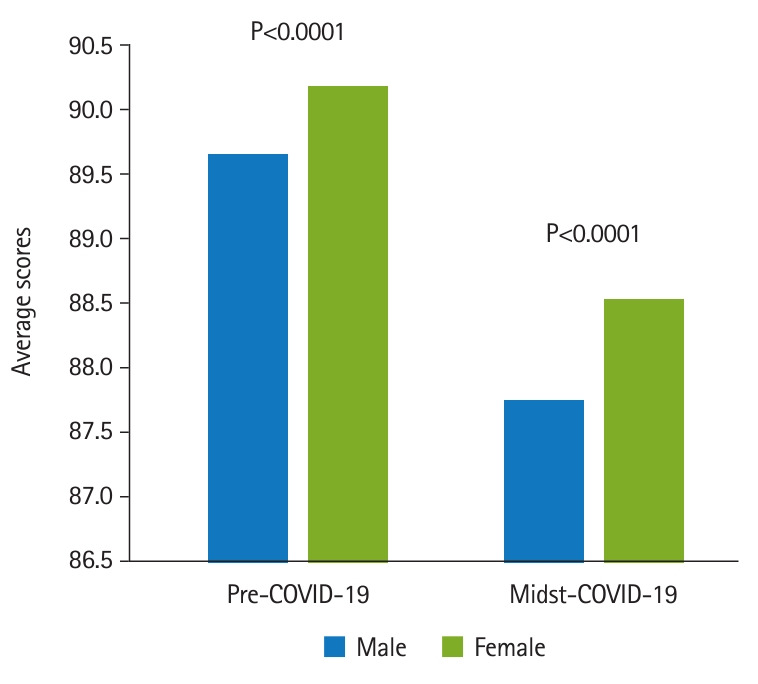
Female attending physician grading: male students versus female students. Pre-COVID-19 male students received an average of 89.68–3.62, and the female students received an average of 90.22–3.93 (Cohen’s d=0.10, P<0.0001). The midst-COVID-19 male students received an average of 87.76–3.67 and the female students received an average of 88.54–3.51 (Cohen’s d=0.15, P<0.0001). COVID-19, coronavirus disease 2019.

Fig. 2 shows that in the COVID-19-affected specialties, the averages and standard deviations were 90.14±3.55 (n=1,434) for pre-COVID-19 group and 88.51±3.52 (n=598) for the midst-COVID-19 group (Cohen’s d=0.46, P<0.0001). In other specialties, the averages and standard deviations were 90.06±3.58 (n=10,494) for pre-COVID-19 group and 88.32±3.68 (n=4,418) for the midst-COVID-19 group (Cohen’s d=0.48, P<0.0001). The attending physicians were divided into groups of 699 male and 219 female physicians. The students comprised 837 males and 485 females. It was found that the male students’ scores (89.33–3.68) were significantly lower than the female students’ scores (89.99–3.66) (Cohen’s d=0.18, P=0.0017). Students’ scores were analyzed in terms of the attending physician’s gender. As shown in Fig. 3, there was a significant difference in the average scores given by male attending physicians to male and female students before the COVID-19 pandemic. Male students received an average of 89.61±3.65, while female students received an average of 90.32±3.94 (Cohen’s d=0.19, P<0.0001). The midst-COVID-19 male students received an average of 87.94–3.69 and the female students received an average of 88.61–3.79 (Cohen’s d=0.13, P<0.0001). Fig. 4 shows that within the female attending physician group, pre-COVID-19 male students received an average of 89.68–3.62, and female students received an average of 90.22–3.93 (Cohen’s d=0.10, P<0.0001). The midst-COVID-19 male students received 87.76–3.67 and the female students received 88.54–3.51 (Cohen’s d=0.22, P<0.0001).

GEE analysis was conducted to evaluate the impact of student’s age (R^2^=0.2509, unstandardized beta coefficient=-0.03, standard error [SE]=0.02, P=0.1278), student’s gender (female) (unstandardized beta coefficient=1.10, SE=0.20, P<0.0001), attending physician’s gender (female) (unstandardized beta coefficient=-0.19, SE=0.08, P=0.0184), COVID-19-affected specialties (unstandardized beta coefficient=0.26, SE=0.11, P=0.0145), and COVID-19 pandemic (unstandardized beta coefficient=-1.99, SE=0.13, P<0.0001) on students’ scores (Table 2). Raw response data are available in Dataset 1.

## Discussion

### Key results

In this study, we found that within COVID-19-affected specialties, the midst-COVID-19 score was significantly lower than the pre-COVID-19 score. Regarding the other specialties, the midst-COVID-19 score was also significantly lower than the midst-COVID-19 score. In terms of gender, male students’ scores were significantly lower than female students’ scores. GEE revealed that the COVID-19 pandemic, COVID-19-affected specialties, student’s gender, and attending physician’s gender were independent factors affecting student performance scores. Only student’s gender positively influenced the students’ scores. The COVID-19 pandemic negatively influenced students’ scores.

### Interpretation

This study revealed a decline in the clinical performance of students trained in COVID-19-affected departments with similar or larger patient populations prior to the pandemic, in contrast to the learning-by-doing theory. The departments most affected by the viral pandemic were the emergency, pulmonary, and infectious medicine departments, primarily due to disease progression. This study consistently revealed: (1) a decline in students’ clinical scores during COVID-19 across specialties and genders, and (2) female students outperforming male students regardless of the COVID-19 pandemic. These results could be attributed to the reduced number of both inpatient and outpatient populations and changes in healthcare diversity during the pandemic. In addition, the elevated risk of nosocomial infection and high fatality rate in vulnerable groups may prevent patients from seeking routine medical care. The lack of patient diversity and cases has affected students’ clinical participation and limited their first-hand experiences. Emic descriptions have been shown to be more memorable because they portray higher imagery values, which in turn improve and solidify knowledge. However, a decline in first-hand experiences may lead to poorer memory of what is learned. For the sake of medical students’ safety, since the beginning of COVID-19, most medical schools have minimized direct patient care. For example, Taipei Veterans General Hospital implemented a transition from conventional education to virtual classes and limited student participation to reduce the risk of transmission. This study showed that students performed worse clinically after the start of the pandemic, and the difficulties encountered with virtual learning may, in part, be the reason for this decline. COVID-19 has affected all aspects of the medical industry. However, gender roles may also affect adaptive learning. Our study showed that female students consistently received higher scores in clinical settings than their male counterparts, regardless of the COVID-19 pandemic status. This might be because women perform better in interpersonal settings. The underlying gender characteristics could have played a role in the students’ clinical performance in this study.

### Comparison with previous studies

Severely infected COVID-19 patients presented with acute respiratory distress syndrome and respiratory failure, thereby placing a high priority on infectious disease physicians and pulmonologists during the pandemic, as in China [7]. COVID-19 created concerns regarding the spread of contagion and led to public social distancing, which inevitably resulted in the deferment and cancellation of elective procedures in Taiwan [8]. Many studies have shown a reduction in hospital inpatient activity during the peak of COVID-19 infections in the United States [9,10]. One study reported a 39% decline in medical access after the COVID-19 outbreak in a community hospital in Taiwan [5]. The learning-by-doing theory states that students better adapt and learn through a hands-on approach to their environment in all countries [11]. As the flow of critical patients greatly increased, emergency departments became overwhelmed with potentially infected cases [12]. Attending and resident physicians were reallocated to critical areas to care for increased cases of acute patients in hospitals [13]. This led to a lack of teaching time, underscoring the need for instruction for medical students. In addition, courses have been altered from in-person teaching to mostly online learning, regardless of the number of patients in the departments, which in turn may interfere with students’ learning capabilities because of a lack of social interaction and lower motivation [2]. In-person lectures are either pre-recorded or live-streamed using online meeting applications. Technology allows the convenience of virtual learning for medical curriculum delivery, case presentations, virtual patient care, testing, and interviews to be delivered to groups or individuals, as reported in the United States [14]. Online teaching allows both group and individual settings, an easy process of students’ interaction in real life, and face-to-face sessions from any location. Although technology provides convenience, personal connections between faculty and students can be challenging in virtual courses. Students may report lack of interaction with instructors, response time deficiencies, and absence of traditional classroom socialization with their peers [2]. In addition, students may be prone to distractions because of stilted interactions. Many clinical practice studies have shown that women performed better under uncertainty and during stressful periods than men in clinical practice [6]. However, our previous study found that male students performed better than female students on standardized written examinations, while female students performed better than male students in various clerkships; additionally, men performed better with knowledge-based content, whereas women seemed more at ease in clinical environments [15].

### Limitations/generalizability

While this study sheds light on the impact of COVID-19 on the clinical performance of Taiwanese medical students, several limitations must be considered. The findings may not be universally applicable, as the study is confined to the context of Taiwan, and differences in healthcare systems, educational structures, and pandemic responses could affect the generalizability of the results to other regions. It is essential to acknowledge that the absence of admission score data of the students limits the depth of the comparative analysis, potentially impacting the comprehensive interpretation of the results. The reliance on online standardized evaluation systems introduces a potential limitation, as these systems may not fully capture the complex and dynamic nature of clinical skills assessment. The study’s temporal scope, covering the period from 2018 to mid-2021, may not fully capture the evolving nature of the pandemic and the subsequent adaptations in medical education. Additionally, the categorization of specialties into COVID-19-affected and other specialties oversimplifies the intricacies within each category, potentially overlooking the unique challenges faced by specific medical disciplines. Furthermore, the study’s focus on specific variables, such as gender and the presence of female attending physicians, may not account for the multifaceted factors influencing medical student performance during the pandemic. Consequently, caution should be exercised when generalizing these findings to diverse global contexts and healthcare settings.

### Suggestion

We believe that our experience can serve as a guide for future studies on avoiding the disruption of traditional education routines, investigating resilient medical education models, prioritizing the integration of online evaluation systems, and considering gender-related factors, for future medical professionals during pandemics.

### Conclusions

COVID-19 has altered medical curricula worldwide. The present study concluded that COVID-19 has negatively affected medical students’ clinical performance, regardless of specialty. In addition, traditional in-person teaching has transitioned to virtual learning. The extended results showed that female students performed better than male students, both before and during the pandemic. This conclusion could be attributed to gender differences. Overall, this study shows that scores declined due to COVID-19, and the findings may be adapted to better-suited medical curricula that address medical education influenced by the pandemic.

## Figures and Tables

**Figure f5-jeehp-20-37:**
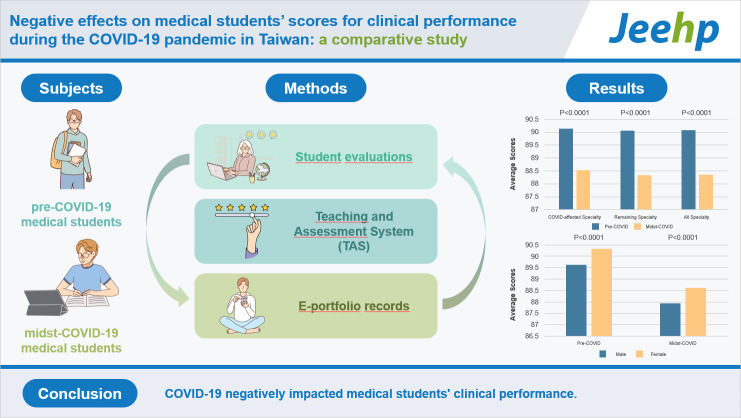


**Table 1.** Baseline characteristics of the participants

Baseline characteristics	Pre-COVID-19	Midst-COVID-19	P-value
Students	830 (62.78)	492 (37.22)	
Male	526 (63.37)	311 (63.21)	0.9529
Age (yr)	24.68±2.39	24.55±2.18	0.3478
Teachers	475 (51.74)	443 (48.26)	
Male	365 (76.84)	334 (75.40)	0.3288
Scores			
COVID-19-affected specialties	1,434 (12.02)	598 (11.92)	0.8545
Other specialties	10,494 (87.98)	4,418 (88.08)	
Total no. of scores	11,928 (100.00)	5,016 (100.00)	

Values are presented as number (%) or mean±standard deviation. COVID-19, coronavirus disease 2019.

**Table 2.** Generalized estimating equations for confounding variables

Score predictors (R^2^=0.2509)	Unstandardized coefficients		Standardized coefficients beta	z-value	P-value
	Beta	Standard error			
Student’s age	-0.03	0.02	-0.02	-1.52	0.1278
Student’s gender (female)	1.10	0.20	0.14	5.52	<0.0001
Attending physician’s gender (female)	-0.19	0.08	-0.02	-2.44	0.0145
COVID-19-affected specialties	0.26	0.11	0.02	2.36	0.0184
COVID-19 pandemic	-1.99	0.13	-0.25	-15.74	<0.0001

COVID-19, coronavirus disease 2019.

## Footnotes

**Data availability**
Data files are available from Harvard Dataverse: https://doi.org/10.7910/DVN/AS90CV Dataset 1. Raw score data of examinees for clinical performance.

## Supplementary materials

Supplement 1. Audio recording of the abstract.
